# Supplementary material for: A Unified Formulation of k-Means, Fuzzy c-Means and Gaussian Mixture Model by the Kolmogorov–Nagumo Average
Source: Entropy (Basel). 2021 Apr 24;23(5):518. doi: 10.3390/e23050518 (PMC8145026; doi:10.3390/e23050518)
Supplement: Supplementary file 1 [file entropy-23-00518-s001.zip › supplementary/supplementary(2021.4.20).pdf]

Supporting information for "A unified formulation of k-means, fuzzy c-means and Gaussian mixture model by Kolmogorov-Nagumo average"

Osamu Komori
Seikei University
Kichijoji Kitamachi, Musashino, Tokyo 180-8633, Japan
Shinto Eguchi
The Institute of Statistical Mathematics
Midori-cho 10-3, Tachikawa Tokyo 190-8562, Japan

A Notations of methods and characteristics of benchmark datasets

Table S 1: Summary of clustering methods

$\tau \setminus \beta$	0	0.5	1
0.5	Gaussian	GP	Pareto
1	Gk_1	GPkf_1	Pf_1
10	Gk_{10}	GPkf_{10}	Pf_{10}
100	Gk_{100}	GPkf_{100}	Pf_{100}
∞	k-means	kf	fuzzy c-means

Table S 2: Sample sizes, the number of clusters and dimensions of benchmark datasets

Dataset	Flame	Compound	D31	Aggregation	Glass	Jain	Pathbased	Spiral	R15	Dim1024	A3	Unbalance	S1,S2,S3 [a]	Birch1,Birch2 [a]
Sample size (n)	240	399	3100	788	214	373	300	312	600	1024	7500	6500	5000	1000
Clusters (K)	2	6	31	7	7	2	3	3	15	16	50	8	15	100
Dimension (d)	2	2	2	2	9	2	2	2	2	1024	2	2	2	2

a S1, S2 and S3 have Gaussian clusters with different degrees of cluster overlap. Birch1 has clusters in a regular grid structure; Birch2 has clusters at a sine curve. Birch1 and Birch2 are subsampled to have small sample sizes.

- See http://cs.uef.fi/sipu/datasets/ for more details.

B Results of benchmark data with $\Sigma_k \neq I$ in Pareto clustering

Table S 3: The result of Purity ($\Sigma_k \neq I$)

Data	k-means	fuzzy-c	GP	Pareto	Gk_1	$GPkf_1$	Pf_1	Gk_{10}	$GPkf_{10}$	Pf_{10}	Gk_{100}	$GPkf_{100}$	Pf_{100}	PAM	Gaussian
Flame	0.829	**0.854**	0.796	0.808	0.771	0.796	0.808	0.771	0.796	0.808	0.771	0.796	0.808	0.85	0.746
Compound	0.789	0.789	0.782	0.767	0.802	0.782	0.767	0.802	0.784	0.767	0.802	0.784	0.769	0.737	**0.845**
D31	0.756	0.822	0.974	0.974	0.973	0.974	0.974	0.973	0.974	0.974	0.973	0.974	0.974	**0.977**	0.875
Aaggregation	0.939	0.871	0.949	0.952	0.948	0.949	0.952	0.948	0.949	0.951	0.948	0.949	0.951	0.945	**0.956**
Glass	**0.472**	0.439	0.407	0.355	0.411	0.374	0.355	0.407	0.374	0.355	0.397	0.379	0.355	0.449	0.383
Jain	**0.874**	0.869	0.786	0.815	0.759	0.786	0.812	0.759	0.783	0.81	0.759	0.783	0.81	0.855	0.74
Pathbased	0.76	**0.763**	0.743	0.753	0.74	0.743	0.753	0.74	0.743	0.753	0.74	0.743	0.753	0.757	0.707
Spiral	0.343	0.34	0.346	0.349	0.34	0.349	0.346	0.34	0.353	0.346	0.34	0.353	0.346	**0.369**	0.349
R15	**0.997**	**0.997**	**0.997**	**0.997**	**0.997**	**0.997**	**0.997**	**0.997**	**0.997**	**0.997**	0.993	**0.997**	**0.997**	**0.997**	**0.997**
Dim1024	0.75	**1**	**1**	**1**	**1**	**1**	**1**	**1**	**1**	**1**	**1**	**1**	**1**	**1**	0.806
a3	0.872	0.853	0.995	0.997	0.994	0.995	0.997	0.994	0.995	0.997	0.994	0.995	0.997	**0.998**	0.586
Unba	**0.969**	0.954	**0.969**	**0.969**	**0.969**	**0.969**	**0.969**	**0.969**	**0.969**	**0.969**	**0.969**	**0.969**	**0.969**	**0.969**	0.954
S1	0.929	0.93	**0.995**	0.994	**0.995**	**0.995**	0.994	**0.995**	**0.995**	0.994	**0.995**	**0.995**	0.994	0.993	**0.995**
S2	0.838	0.907	**0.972**	0.969	0.97	**0.972**	0.969	0.97	**0.972**	0.969	0.97	**0.972**	0.969	0.97	0.967
S3	0.754	0.803	**0.865**	0.858	0.857	**0.865**	0.858	0.857	**0.865**	0.858	0.857	**0.865**	0.858	0.859	0.841
Birch1	0.84	0.813	0.894	0.902	0.891	0.894	0.903	0.89	0.891	0.904	0.89	0.891	0.904	**0.925**	0.643
Birch2	0.755	0.736	0.869	**0.875**	0.867	0.869	0.874	0.865	0.867	0.874	0.865	0.867	0.874	0.855	0.588

Table S 4: The result of F values ($\Sigma_k \neq I$)

Data	k-means	fuzzy-c	GP	Pareto	Gk_1	$GPkf_1$	Pf_1	Gk_{10}	$GPkf_{10}$	Pf_{10}	Gk_{100}	$GPkf_{100}$	Pf_{100}	PAM	Gaussian
Flame	0.832	**0.857**	0.799	0.812	0.774	0.799	0.812	0.774	0.799	0.812	0.774	0.799	0.812	0.853	0.748
Compound	0.622	0.63	0.614	0.606	0.644	0.615	0.606	0.634	0.616	0.606	0.634	0.616	0.609	0.601	**0.719**
D31	0.758	0.789	0.974	0.974	0.973	0.974	0.974	0.973	0.974	0.974	0.973	0.974	0.974	**0.977**	0.877
Aggregation	0.852	0.755	0.863	0.857	0.867	0.864	0.856	0.868	0.864	0.855	0.868	0.864	0.855	0.848	**0.876**
Glass	**0.433**	0.337	0.363	0.375	0.404	0.35	0.375	0.398	0.35	0.375	0.388	0.35	0.375	0.375	0.377
Jain	**0.88**	0.875	0.799	0.826	0.773	0.799	0.824	0.773	0.796	0.821	0.773	0.796	0.821	0.863	0.587
Pathbased	0.721	**0.726**	0.696	0.711	0.691	0.696	0.711	0.691	0.696	0.711	0.691	0.696	0.711	0.716	0.655
Spiral	0.352	0.345	0.346	0.346	0.343	0.348	0.343	0.345	0.349	0.343	0.345	0.349	0.343	**0.377**	0.347
R15	**0.997**	**0.997**	**0.997**	**0.997**	**0.997**	**0.997**	**0.997**	**0.997**	**0.997**	**0.997**	0.993	**0.997**	**0.997**	**0.997**	**0.997**
Dim1024	0.831	**1**	**1**	**1**	**1**	**1**	**1**	**1**	**1**	**1**	**1**	**1**	**1**	**1**	0.853
A3	0.86	0.824	0.995	0.997	0.994	0.995	0.997	0.994	0.995	0.997	0.994	0.995	0.997	**0.998**	0.689
Unba	0.867	0.749	0.778	0.776	0.78	0.779	0.776	0.78	0.778	0.776	0.78	0.778	0.776	0.78	**0.941**
S1	0.944	0.928	**0.995**	0.994	**0.995**	**0.995**	0.994	**0.995**	**0.995**	0.994	**0.995**	**0.995**	0.994	0.993	**0.995**
S2	0.832	0.906	**0.972**	0.969	0.97	**0.972**	0.969	0.97	**0.972**	0.969	0.97	**0.972**	0.969	0.97	0.966
S3	0.758	0.801	**0.864**	0.857	0.856	**0.864**	0.857	0.856	**0.864**	0.857	0.856	**0.864**	0.857	0.858	0.843
Birch1	0.826	0.783	0.885	0.891	0.88	0.885	0.892	0.879	0.882	0.893	0.879	0.882	0.893	**0.912**	0.666
Birch2	0.724	0.715	0.86	**0.863**	0.857	0.86	0.862	0.855	0.858	0.862	0.855	0.858	0.862	0.832	0.645

Table S 5: The result of Centroid index ($\Sigma_k \neq I$)

Data	k-means	fuzzy-c	GP	Pareto	Gk_1	$GPkf_1$	Pf_1	Gk_{10}	$GPkf_{10}$	Pf_{10}	Gk_{100}	$GPkf_{100}$	Pf_{100}	PAM	Gaussian
Flame	**0**	**0**	**0**	**0**	**0**	**0**	**0**	**0**	**0**	**0**	**0**	**0**	**0**	**0**	**0**
Compound	**2**	3	**2**	**2**	**2**	**2**	**2**	**2**	**2**	**2**	**2**	**2**	**2**	**2**	**2**
D31	7	5	**0**	**0**	**0**	**0**	**0**	**0**	**0**	**0**	**0**	**0**	**0**	**0**	3
Aggregation	**1**	2	**1**	**1**	**1**	**1**	**1**	**1**	**1**	**1**	**1**	**1**	**1**	**1**	**1**
Glass	3	3	3	3	3	3	3	2	3	3	2	3	3	4	**1**
Jain	**0**	**0**	**0**	**0**	**0**	**0**	**0**	**0**	**0**	**0**	**0**	**0**	**0**	**0**	1
Pathbased	**1**	**1**	**1**	**1**	**1**	**1**	**1**	**1**	**1**	**1**	**1**	**1**	**1**	**1**	**1**
Spiral	1	1	**0**	**0**	**0**	**0**	**0**	**0**	**0**	**0**	**0**	**0**	**0**	1	**0**
R15	**0**	**0**	**0**	**0**	**0**	**0**	**0**	**0**	**0**	**0**	**0**	**0**	**0**	**0**	**0**
Dim1024	4	**0**	**0**	**0**	**0**	**0**	**0**	**0**	**0**	**0**	**0**	**0**	**0**	**0**	**0**
A3	6	7	**0**	**0**	**0**	**0**	**0**	**0**	**0**	**0**	**0**	**0**	**0**	**0**	18
Unba	**2**	3	**2**	**2**	**2**	**2**	**2**	**2**	**2**	**2**	**2**	**2**	**2**	**2**	3
S1	1	1	**0**	**0**	**0**	**0**	**0**	**0**	**0**	**0**	**0**	**0**	**0**	**0**	**0**
S2	2	1	**0**	**0**	**0**	**0**	**0**	**0**	**0**	**0**	**0**	**0**	**0**	**0**	**0**
S3	2	1	**0**	**0**	**0**	**0**	**0**	**0**	**0**	**0**	**0**	**0**	**0**	**0**	**0**
Birch1	12	18	**4**	**4**	**4**	5	**4**	**4**	5	**4**	**4**	5	**4**	**4**	34
Birch2	23	25	**8**	9	**8**	**8**	9	**8**	9	9	**8**	9	9	9	49

C Results of benchmark data with $\Sigma_k = I$ in Pareto clustering

Table S 6: The result of Purity ($\Sigma_k = I$)

data	k-means	fuzzy-c	GP	Pareto	Gk_1	$GPkf_1$	Pf_1	Gk_{10}	$GPkf_{10}$	Pf_{10}	Gk_{100}	$GPkf_{100}$	Pf_{100}	PAM	Gaussian
Flame	0.829	**0.854**	0.637	0.637	0.771	0.779	0.762	0.758	0.771	0.783	0.758	0.771	0.783	0.85	0.746
Compound	0.789	0.789	0.729	0.737	0.802	0.762	0.739	0.797	0.787	0.757	0.797	0.784	0.764	0.737	**0.845**
D31	0.756	0.822	0.286	0.356	0.498	0.609	0.567	0.965	0.967	0.963	0.972	0.973	0.973	**0.977**	0.875
Aggregation	0.939	0.871	0.954	0.952	0.954	**0.956**	0.952	0.954	**0.956**	0.953	0.954	**0.956**	0.953	0.945	**0.956**
Glass	**0.472**	0.439	0.388	0.355	0.444	0.355	0.355	0.411	0.36	0.355	0.416	0.36	0.355	0.449	0.383
Jain	**0.874**	0.869	0.861	**0.874**	0.855	0.858	0.861	0.842	0.855	0.861	0.842	0.855	0.861	0.855	0.74
Pathbased	0.76	**0.763**	0.657	0.633	0.737	0.64	0.633	0.747	0.74	0.727	0.747	0.74	0.73	0.757	0.707
Spiral	0.343	0.34	**0.513**	0.423	0.372	0.369	0.375	0.362	0.356	0.356	0.362	0.356	0.356	0.369	0.349
R15	**0.997**	**0.997**	0.58	0.55	0.755	0.795	0.74	0.992	0.992	0.992	0.993	0.993	**0.997**	**0.997**	**0.997**
Dim1024	0.75	**1**	**1**	**1**	**1**	**1**	**1**	**1**	**1**	**1**	**1**	**1**	**1**	**1**	0.806
A3	0.872	0.853	0.301	0.274	0.492	0.587	0.554	0.928	0.962	0.95	0.99	0.992	0.992	**0.998**	0.586
Unba	**0.969**	0.954	0.338	0.338	0.67	0.858	0.808	**0.969**	**0.969**	**0.969**	**0.969**	**0.969**	**0.969**	**0.969**	0.954
S1	0.929	0.93	0.61	0.509	0.713	0.772	0.655	0.993	0.994	0.994	0.993	0.994	**0.995**	0.993	**0.995**
S2	0.838	0.907	0.517	0.422	0.568	0.746	0.651	0.966	0.969	0.964	0.966	**0.97**	0.965	**0.97**	0.967
S3	0.754	0.803	0.507	0.469	0.608	0.596	0.538	0.848	0.849	0.844	0.848	0.85	0.845	**0.859**	0.841
Birch1	0.84	0.813	0.324	0.288	0.407	0.437	0.438	0.694	0.711	0.691	0.885	0.892	0.891	**0.925**	0.643
Birch2	0.755	0.736	0.277	0.278	0.269	0.359	0.415	0.393	0.495	0.512	0.749	0.758	0.752	**0.855**	0.588

Table S 7: The result of F values ($\Sigma_k = I$)

data	k-means	fuzzy-c	GP	Pareto	Gk_1	$GPkf_1$	Pf_1	Gk_{10}	$GPkf_{10}$	Pf_{10}	Gk_{100}	$GPkf_{100}$	Pf_{100}	PAM	Gaussian
Flame	0.832	**0.857**	0.644	0.638	0.774	0.783	0.766	0.761	0.774	0.787	0.761	0.774	0.787	0.853	0.748
Compound	0.622	0.63	0.701	0.673	**0.772**	0.762	0.726	0.628	0.618	0.592	0.633	0.62	0.597	0.601	0.719
D31	0.758	0.789	0.35	0.419	0.537	0.645	0.596	0.965	0.967	0.962	0.972	0.973	0.973	**0.977**	0.877
Aggregation	0.852	0.755	0.871	0.865	0.876	0.874	0.868	**0.879**	0.875	0.869	**0.879**	0.875	0.869	0.848	0.876
Glass	**0.433**	0.337	0.356	0.375	0.38	0.359	0.375	0.336	0.357	0.375	0.339	0.358	0.375	0.375	0.377
Jain	**0.88**	0.875	0.868	**0.88**	0.863	0.866	0.868	0.851	0.863	0.868	0.851	0.863	0.868	0.863	0.587
Pathbased	0.721	**0.726**	0.65	0.652	0.686	0.656	0.653	0.701	0.691	0.67	0.701	0.691	0.675	0.716	0.655
Spiral	0.352	0.345	**0.512**	0.424	0.372	0.368	0.375	0.365	0.358	0.357	0.365	0.358	0.357	0.377	0.347
R15	**0.997**	**0.997**	0.59	0.582	0.762	0.787	0.732	0.992	0.992	0.992	0.993	0.993	**0.997**	**0.997**	**0.997**
Dim1024	0.831	**1**	**1**	**1**	**1**	**1**	**1**	**1**	**1**	**1**	**1**	**1**	**1**	**1**	0.853
A3	0.86	0.824	0.378	0.35	0.549	0.622	0.589	0.925	0.962	0.949	0.99	0.992	0.992	**0.998**	0.689
Unba	0.867	0.749	0.502	0.5	0.714	0.865	0.806	0.942	0.949	**0.963**	0.778	0.781	0.785	0.78	0.941
S1	0.944	0.928	0.649	0.581	0.752	0.801	0.682	0.993	0.994	0.994	0.993	0.994	**0.995**	0.993	**0.995**
S2	0.832	0.906	0.548	0.478	0.62	0.761	0.669	0.966	0.968	0.964	0.966	0.969	0.965	**0.97**	0.966
S3	0.758	0.801	0.53	0.506	0.619	0.604	0.56	0.846	0.848	0.842	0.846	0.848	0.844	**0.858**	0.843
Birch1	0.826	0.783	0.353	0.32	0.424	0.452	0.464	0.69	0.708	0.68	0.875	0.883	0.881	**0.912**	0.666
Birch2	0.724	0.715	0.317	0.315	0.299	0.395	0.464	0.439	0.53	0.551	0.759	0.77	0.747	**0.832**	0.645

Table S 8: The result of Centroid index ($\Sigma_k = I$)

data	k-means	fuzzy-c	GP	Pareto	Gk_1	$GPkf_1$	Pf_1	Gk_{10}	$GPkf_{10}$	Pf_{10}	Gk_{100}	$GPkf_{100}$	Pf_{100}	PAM	Gaussian
Flame	**0**	**0**	1	1	**0**	**0**	**0**	**0**	**0**	**0**	**0**	**0**	**0**	**0**	**0**
Compound	**2**	**2**	3	3	3	3	**2**	**2**	**2**	**2**	**2**	**2**	**2**	**2**	**2**
D31	7	6	28	29	21	20	23	2	2	3	**1**	**1**	**1**	**1**	4
Aggregation	**1**	2	**1**	**1**	**1**	**1**	**1**	**1**	**1**	**1**	**1**	**1**	**1**	**1**	**1**
Glass	3	**2**	**2**	4	**2**	**2**	3	**2**	**2**	**2**	**2**	**2**	**2**	3	**2**
Jain	**0**	**0**	**0**	**0**	**0**	**0**	**0**	**0**	**0**	**0**	**0**	**0**	**0**	**0**	**0**
Pathbased	**1**	**1**	2	2	**1**	**1**	**1**	**1**	**1**	**1**	**1**	**1**	**1**	**1**	**1**
Spiral	2	2	2	2	2	2	2	2	2	2	2	2	**1**	2	**1**
R15	**0**	**0**	14	14	7	7	7	**0**	**0**	**0**	**0**	**0**	**0**	**0**	**0**
Dim1024	4	**0**	**0**	**0**	**0**	**0**	**0**	**0**	**0**	**0**	**0**	**0**	**0**	**0**	**0**
A3	6	7	46	47	38	34	39	7	7	10	**0**	1	1	**0**	18
Unba	**2**	3	5	5	3	3	3	**2**	**2**	**2**	**2**	**2**	**2**	**2**	3
S1	1	1	12	12	8	9	9	**0**	**0**	**0**	**0**	**0**	**0**	**0**	**0**
S2	2	1	11	13	8	8	9	**0**	**0**	**0**	**0**	**0**	**0**	**0**	**0**
S3	4	**1**	13	13	6	7	9	2	2	2	2	2	2	2	2
Birch1	**16**	18	93	96	81	75	72	38	38	39	4	4	6	6	34
Birch2	27	27	93	93	91	86	84	51	54	56	21	23	22	**14**	49

D Tuning of parameters τ and β

We discuss a sample based selection of tuning parameters β and τ, in which the estimator for the vector μ of cluster centers depends on β and τ as

$$\hat{\mu}_{\tau,\beta} = \underset{\mu}{\operatorname{argmin}}\, L_{\tau,\beta}(\mu). \tag{S.1}$$

Here we consider $L_{\tau,\beta}(\mu)$ instead of $L_{\tau,\beta}(\theta)$ for simplicity. We fix the loss function to assess $\hat{\mu}_{\tau,\beta}$, so that the loss function is independent of β and τ. For example, we adopt the loss function of fuzzy c-means as

$$L_0(\mu) = \sum_{i=1}^{n} \Big(\frac{1}{K} \sum_{k=1}^{K} \|x_i - \mu_k\|^{\frac{2}{1-m_0}} \Big)^{1-m_0}, \tag{S.2}$$

where m_0 is a fixed constant. Henceforth, we call $L_0(\mu)$ the anchor loss function. There arises a serious problem due to overlearning if we naively take the argument to minimize $L_0(\hat{\mu}_{\tau,\beta})$ in (β, τ) for the selection. In fact, we employ the same data set to evaluate $L_0(\mu)$ and $\hat{\mu}_{\tau,\beta}$, in which the anchor loss function $L_0(\mu)$ plugged-in $\mu = \hat{\mu}_{\tau,\beta}$ is written by

$$L_0(\hat{\mu}_{\tau,\beta}) = \sum_{i=1}^{n} \Big(\frac{1}{K} \sum_{k=1}^{K} \|x_i - \hat{\mu}_{\tau,\beta k}\|^{\frac{2}{1-m_0}} \Big)^{1-m_0}, \tag{S.3}$$

which gives bias from the dependence between x_i and $\hat{\mu}_{\tau,\beta}$. If $\{x_i\}$ in $(S.3)$ are independent of the data set used in $(S.1)$, a biased aspect would not occur. We build on a method based on the leave-one-out cross validation with a low computational cost. For simplicity in the subsequent discussion we write

$$L_0(\mu) = \sum_{i=1}^{n} \ell_0(x_i, \mu) \tag{S.4}$$

and

$$L_{\tau,\beta}(\mu) = \sum_{i=1}^{n} \ell_{\tau,\beta}(x_i, \mu), \tag{S.5}$$

where

$$\ell_0(x, \mu) = \Big(\frac{1}{K} \sum_{k=1}^{K} \|x - \mu_k\|^{\frac{2}{1-m_0}} \Big)^{1-m_0} \tag{S.6}$$

$$\ell_{\tau,\beta}(x, \mu) = \frac{1}{\tau\beta} \Big[\Big\{ \frac{1}{K} \sum_{k=1}^{K} (1 + \tau\beta\|x - \mu_k\|^2)^{-\frac{1}{\beta}} \Big\}^{-\beta} - 1 \Big]. \tag{S.7}$$

Then the leave-one-one statistic is given by

$$\mathrm{LOO}(\beta, \tau) = \sum_{i=1}^{n} \ell_0(x_i, \mu_{\tau,\beta}^{(-i)}), \tag{S.8}$$

where

$$\mu_{\tau,\beta}^{(-i)} = \underset{\mu}{\operatorname{argmin}} \sum_{j \neq i}^{n} \ell_{\tau,\beta}(x_j, \mu). \tag{S.9}$$

Thus, $\mathrm{LOO}(\beta,\tau)$ prevents from overlearning because x_i and $\mu_{\tau,\beta}^{(-i)}$ are statistically independent. However it incurs a high cost to compute the validated estimates $\mu_{\tau,\beta}^{(-i)}$ as follows. Here, we consider an approximated analogue for $\mathrm{LOO}(\beta,\tau)$. By the definition of $\mu_{\tau,\beta}^{(-i)}$,

$$\sum_{j\neq i}\frac{\partial}{\partial\mu}\ell_{\tau,\beta}(x_j,\mu_{\tau,\beta}^{(-i)})=0, \tag{S.10}$$

which means

$$\sum_{j=1}^{n}\frac{\partial}{\partial\mu}\ell_{\tau,\beta}(x_j,\mu_{\tau,\beta}^{(-i)})=\frac{\partial}{\partial\mu}\ell_{\tau,\beta}(x_i,\mu_{\tau,\beta}^{(-i)}), \tag{S.11}$$

and is approximately given by

$$\sum_{j=1}^{n}\frac{\partial^2}{\partial\mu\partial\mu^\top}\ell_{\tau,\beta}(x_j,\hat\mu_{\tau,\beta})(\hat\mu_{\tau,\beta}-\mu_{\tau,\beta}^{(-i)})=\frac{\partial}{\partial\mu}\ell_{\tau,\beta}(x_i,\mu_{\tau,\beta}^{(-i)})+o_{\mathrm{P}}(1). \tag{S.12}$$

Therefore we conclude that

$$\hat\mu_{\tau,\beta}-\mu_{\tau,\beta}^{(-i)}=h_{\beta.\tau}(x_i)+o_{\mathrm{P}}(n^{-1}). \tag{S.13}$$

where

$$h_{\beta.\tau}(x_i)=\Big\{\sum_{j=1}^{n}\frac{\partial^2}{\partial\mu\partial\mu^\top}\ell_{\tau,\beta}(x_j,\hat\mu_{\tau,\beta})\Big\}^{-1}\frac{\partial}{\partial\mu}\ell_{\tau,\beta}(x_i,\hat\mu_{\tau,\beta}). \tag{S.14}$$

In accordance with this, we have an approximation for $\mathrm{LOO}(\beta,\tau)$ by

$$\widetilde{\mathrm{LOO}}(\beta,\tau)=\sum_{i=1}^{n}\ell_0(x_i,\hat\mu_{\tau,\beta}-h_{\beta.\tau}(x_i)) \tag{S.15}$$

and the expanded version

$$\widetilde{\mathrm{LOO}}_1(\beta,\tau)=L_0(\hat\mu_{\tau,\beta})-\sum_{i=1}^{n}\frac{\partial}{\partial\mu^\top}\ell_0(x_i,\hat\mu_{\tau,\beta})h_{\beta.\tau}(x_i). \tag{S.16}$$

Hence, we propose a selection method by

$$(\hat\beta,\hat\tau)=\underset{\tau,\beta}{\mathrm{argmin}}\,\widetilde{\mathrm{LOO}}(\beta,\tau). \tag{S.17}$$

$$\begin{aligned}
\ell_{\tau,\beta}(x,\mu) &= \frac{1}{\tau\beta}\Big[\Big\{\frac{1}{K}\sum_{k=1}^{K}(1+\tau\beta\|x-\mu_k\|^2)^{-\frac{1}{\beta}}\Big\}^{-\beta}-1\Big] &\text{(S.18)}\\
&= \frac{1}{\tau\beta}\Big[\Big\{\frac{1}{K}\sum_{k=1}^{K}w(x,\mu_k)\Big\}^{-\beta}-1\Big] &\text{(S.19)}\\
&= \frac{1}{\tau\beta}(\overline{w}(x,\mu)^{-\beta}-1), &\text{(S.20)}
\end{aligned}$$

where

$$w(x, \mu_k) = (1 + \tau\beta\|x - \mu_k\|^2)^{-\frac{1}{\beta}} \tag{S.21}$$

$$\overline{w}(x, \mu) = \frac{1}{K}\sum_{k=1}^{K} w(x, \mu_k). \tag{S.22}$$

Here we have

$$\frac{\partial}{\partial\mu_k}\ell_{\tau,\beta}(x, \mu) = \frac{1}{\tau\beta}(-\beta)\overline{w}(x, \mu)^{-\beta-1}\frac{1}{K}\frac{\partial}{\partial\mu_k}w(x, \mu_k) \tag{S.23}$$

$$= -\frac{1}{\tau K}\overline{w}(x, \mu)^{-\beta-1}(-\frac{1}{\beta})(1 + \tau\beta\|x - \mu_k\|^2)^{-\frac{1+\beta}{\beta}}2\tau\beta(x - \mu_k)(-1) \tag{S.24}$$

$$= -\frac{2}{K}\overline{w}(x, \mu)^{-\beta-1}w(x, \mu_k)^{1+\beta}(x - \mu_k) \tag{S.25}$$

$$= -\frac{2}{K}\left(\frac{w(x, \mu_k)}{\frac{1}{K}\sum_{i=1}^{K} w(x, \mu_k)}\right)^{1+\beta}(x - \mu_k) \tag{S.26}$$

$$= -2K^{\beta}\left(\frac{w(x, \mu_k)}{\sum_{i=1}^{K} w(x, \mu_k)}\right)^{1+\beta}(x - \mu_k) \tag{S.27}$$

For $k \neq m$ we have

$$\frac{\partial}{\partial\mu_k\partial\mu_m^{\top}}\ell_{\tau,\beta}(x, \mu) \tag{S.28}$$

$$= -\frac{2}{K}w(x, \mu_k)^{1+\beta}(x - \mu_k)\frac{\partial}{\partial\mu_m^{\top}}\overline{w}(x, \mu)^{-\beta-1} + \frac{2}{K}\overline{w}(x, \mu)^{-\beta-1}\frac{\partial}{\partial\mu_m^{\top}}\{w(x, \mu_k)^{1+\beta}(x - \mu_k)\} \tag{S.29}$$

$$= -\frac{2}{K}w(x, \mu_k)^{1+\beta}(x - \mu_k)\frac{\partial}{\partial\mu_m^{\top}}\overline{w}(x, \mu)^{-\beta-1} \tag{S.30}$$

$$= -\frac{2}{K}w(x, \mu_k)^{1+\beta}(x - \mu_k)(-\beta - 1)\overline{w}(x, \mu)^{-\beta-2}\frac{1}{K}\frac{\partial}{\partial\mu_m^{\top}}w(x, \mu_m) \tag{S.31}$$

$$= \frac{2(1 + \beta)}{K^2}w(x, \mu_k)^{1+\beta}\overline{w}(x, \mu)^{-\beta-2}(x - \mu_k)(-\frac{1}{\beta})(1 + \tau\beta\|x - \mu_m\|^2)^{-\frac{1+\beta}{\beta}}2\tau\beta(x - \mu_m)^{\top}(-1) \tag{S.32}$$

$$= \frac{4\tau(1 + \beta)}{K^2}\overline{w}(x, \mu)^{-\beta-2}w(x, \mu_k)^{1+\beta}w(x, \mu_m)^{1+\beta}(x - \mu_k)(x - \mu_m)^{\top} \tag{S.33}$$

And we have

$$\frac{\partial}{\partial\mu_k^{\top}}\{w(x, \mu_k)^{1+\beta}(x - \mu_k)\} \tag{S.34}$$

$$= (x - \mu_k)(-\frac{1 + \beta}{\beta})(1 + \tau\beta\|x - \mu_k\|^2)^{-\frac{1+2\beta}{\beta}}2\tau\beta(x - \mu_k)^{\top}(-1) + w(x, \mu_k)^{1+\beta}I(-1) \tag{S.35}$$

$$= 2\tau(1 + \beta)w(x, \mu_k)^{1+2\beta}(x - \mu_k)(x - \mu_k)^{\top} - w(x, \mu_k)^{1+\beta}I \tag{S.36}$$

Hence for $k = m$, we have

$$\frac{\partial}{\partial \mu_k \partial \mu_k^\top} \ell_{\tau,\beta}(x,\mu) = \frac{4\tau(1+\beta)}{K^2} \overline{w}(x,\mu)^{-\beta-2} w(x,\mu_k)^{2(1+\beta)} (x-\mu_k)(x-\mu_k)^\top \tag{S.37}$$

$$+ \frac{2}{K} \overline{w}(x,\mu)^{-\beta-1} \Big\{ 2\tau(1+\beta) w(x,\mu_k)^{1+2\beta} (x-\mu_k)(x-\mu_k)^\top - w(x,\mu_k)^{1+\beta} I \Big\} \tag{S.38}$$

Hence we have

$$\frac{\partial}{\partial \mu_k \partial \mu_m^\top} \ell_{\tau,\beta}(x,\mu) \tag{S.39}$$

$$= \frac{4\tau(1+\beta)}{K^2} \overline{w}(x,\mu)^{-\beta-2} w(x,\mu_k)^{1+\beta} \Big\{ w(x,\mu_m)^{1+\beta} + \delta_{km} K \overline{w}(x,\mu) w(x,\mu_m)^{\beta} \Big\} (x-\mu_k)(x-\mu_m)^\top \tag{S.40}$$

$$- \frac{2}{K} \delta_{km} \overline{w}(x,\mu)^{-\beta-1} w(x,\mu_k)^{1+\beta} I \tag{S.41}$$

E R code of the Pareto clustering

Arguments

- x: data matrix of $n \times d$
- tau, beta: parameters of Pareto clustering τ and β
- iter: iteration step T
- centers: locations of cluster centers of $K \times d$
- Sigma: a list of variance components Σ_k
- pi: a vector of mixing proportions $\pi_1, \ldots, \pi_K$

R code

```
function(x,tau,beta,iter=100,centers,Sigma,pi) {
  epsilon= .Machine$double.xmin*1000
  Max= .Machine$double.xmax/1000
  n=dim(x)[1]
  d=dim(x)[2]
  det_k=1
  if(2-d*beta<=0)
    beta=1.99/d
  K=dim(centers)[1]
```

```
  for(i in 1:iter){
    centers.old=centers
    q_numerator=NULL
    s_numerator=NULL
    for(k in 1:K){
       z=t(t(x)-centers[k,])
       Sigma[[k]]=0.95*Sigma[[k]]+0.05*diag(max(diag(Sigma[[k]]),epsilon),d)
       det_k=abs(det(Sigma[[k]]))
       w=det_k^(-1/2)*(1+beta*tau*diag(z%*%ginv(Sigma[[k]])%*%t(z)))^(-1/beta)
       q_numerator=cbind(q_numerator,pi[k]*w)
    }
    q_numerator[q_numerator==Inf]=Max
    q_numerator[q_numerator==0]=epsilon
    q=q_numerator/apply(q_numerator,1,sum)
    q[q==Inf]=Max
    q[q==0]=epsilon
    pi_numerator=numeric(0)
    for(k in 1:K){
      centers[k,]=apply(q[,k]^(1+beta)*x,2,sum)/sum(q[,k]^(1+beta))
      z=t(t(x)-centers[k,])
      Sigma[[k]]=tau*(2-p*beta)*t(q[,k]^(1+beta)*z)%*%z/sum(q[,k]^(1+beta))
      Sigma[[k]]=0.95*Sigma[[k]]+0.05*diag(max(diag(Sigma[[k]]),epsilon),d)
      det_k=abs(det(Sigma[[k]]))
      B=det_k^(beta/2)*(1+beta*tau*diag(z%*%ginv(Sigma[[k]])%*%t(z)))
      pi_numerator[k]= (  sum(q[,k]^(1+beta)*B)  )^(1/(1+beta))
    }
    pi_numerator[pi_numerator==Inf]=Max
    pi_numerator[pi_numerator==0]=epsilon
    pi=pi_numerator/(sum(pi_numerator))
    Sa=mean((centers-centers.old)^2)
    if(Sa<10^(-10))
          break
  }
  return(list(centers=centers,Sigma=Sigma,pi=pi))
}
```
